# Molecular Screening of Virulence Genes in Extraintestinal Pathogenic *Escherichia coli* Isolated from Human Blood Culture in Brazil

**DOI:** 10.1155/2014/465054

**Published:** 2014-04-15

**Authors:** Vanessa L. Koga, Geizecler Tomazetto, Paula S. Cyoia, Meiriele S. Neves, Marilda C. Vidotto, Gerson Nakazato, Renata K. T. Kobayashi

**Affiliations:** Laboratory of Basic and Applied Bacteriology, Department of Microbiology, Universidade Estadual de Londrina, Rodovia Celso Garcia Cid, 86051-980 Londrina, PR, Brazil

## Abstract

Extraintestinal pathogenic *Escherichia coli* (ExPEC) is one of the main etiological agents of bloodstream infections caused by Gram-negative bacilli. In the present study, 20 *E. coli* isolates from human hemocultures were characterized to identify genetic features associated with virulence (pathogenicity islands markers, phylogenetic group, virulence genes, plasmid profiles, and conjugative plasmids) and these results were compared with commensal isolates. The most prevalent pathogenicity island, in strains from hemoculture, were PAI IV_536_, described by many researchers as a stable island in enterobacteria. Among virulence genes, *iut*A gene was found more frequently and this gene enconding the aerobactin siderophore receptor. According to the phylogenetic classification, group B2 was the most commonly found. Additionally, through plasmid analysis, 14 isolates showed plasmids and 3 of these were shown to be conjugative. Although in stool samples of healthy people the presence of commensal strains is common, human intestinal tract may serve as a reservoir for ExPEC.

## 1. Introduction

*Escherichia coli *is one of the most common microorganisms of the human intestinal microbiota. However, a small percentage of* E. coli* is capable of causing extraintestinal infections (extraintestinal pathogenic* Escherichia coli*—ExPEC), and these ExPECs are considered some of the main etiological agents of bacteremia caused by Gram-negative bacilli [1, 2]. According to the phylogenetic classification, ExPECs typically belong to group B2 and less commonly to group D, whereas commensal intestinal strains belong to group A or B1 [3]. ExPEC pathogenicity is due to the presence of genes, located on plasmids or chromosomes that encode virulence factors. When present on the chromosome, these genes are typically found in specific regions called pathogenicity islands (PAI). Given that the severity of bacterial infections is often due to the genetic features of the pathogenic agent and that few studies have investigated genetic aspects of ExPEC isolates from bacteremia in Brazil [4, 5], the aim of the present study was to characterize 20* E. coli* isolates from human hemocultures for genetic features associated with ExPEC. The investigation was based on the screening for PAI associated sequences, determination of phylogenetic group, genotypic identification of the major virulence factors of ExPEC, and plasmid analyses, and these results compared with commensal strains. This information may help us better understand the pathogenesis of these bacteria.

## 2. Materials and Methods

### 2.1. Bacterial Isolates

To perform the study, 20* E. coli* strains isolated from human hemocultures were kindly provided by Professor Marilda C. Vidotto (Brazil) [6] and 51* E. coli* strains were obtained from stools of 19 healthy Brazilians. The strains were stored in brain heart infusion with 20% glycerol at −20°C. The polymerase chain reaction (PCR) was used for the genetic characterization of PAI associated sequences, presence of virulence genes, and for phylogenetic classification. PCR amplicons were visualized on 1.0% agarose gels stained with GelRed (Biotium). After gel electrophoresis the images were captured using Image Capture Systems (LPixImageHE).

### 2.2. Detection of PAI Markers

The presence of sequences associated with seven different PAIs, previously characterized in uropathogenic* E. coli* (UPEC), was determined (PAI I_536_, II_536_, IV_536_, I_CFT073_, II_CFT073_, I_J96_ and II_J96_) [7] (Table 1).

This PCR contained 1 U Taq DNA polymerase (Invitrogen) in 2x PCR buffer (Invitrogen), 0.2 mM of each dNTP, 2.5 mM MgCl_2_, and 20 pmol/*μ*L of each primer (Table 1). The program consisted of 94°C for 5 min, followed by 30 cycles of 94°C for 1 min, 55°C for 1 min, and 72°C for 1 min, with a final extension step at 72°C for 10 min [7]. The positive control used in the PCR was J96.

### 2.3. Phylogenetic Classification

Phylogenetic classification showed that the* E. coli* strains belonged to four groups (A, B1, B2, or D) based on the presence of the* chu*A and* yja*A genes and the DNA fragment (TSPE4.C2). This PCR contained 1.25 U Taq DNA polymerase (Invitrogen) in 1x PCR buffer (Invitrogen), 20 pmol of each dNTP, 2.5 mM MgCl_2_, and 1 *μ*M of each primer (Table 1). The program of PCR consisted of 94°C for 4 min, followed by 30 cycles of 94°C for 5 seg and 54°C for 10 seg, with a final extension step at 72°C for 5 min [3].

### 2.4. Virulence Factors Genes

The pathogenicity of* E. coli *is associated with the presence of virulence factors that can be encoded by chromosomal and plasmid genes, and thus 19 genes encoding virulence factors were investigated. The genes selected were specific for hemolysins (*hly*A and* hly*F), cytotoxic necrotizing factors (*cnf*1 and* cnf*2), colicin V (*cva*C), aerobactin (*iut*A), yersiniabactin (*fyu*A), salmochelin (*iro*N), P-fimbriae (*pap*C and* pap*G), S-fimbrial adhesin (*sfa*A and* sfa*S), afimbrial adhesin (*afa*), serum resistance (*iss *and* tra*T), brain microvascular endothelium invasion (*ibe*10), K1 capsule (*kps*II and K1), and ompT outer membrane protein (*omp*T) [8–10]. This PCR contained 1.25 U Taq DNA polymerase (Invitrogen) in 1x PCR buffer (Invitrogen), 0.2 mM of each dNTP, 2.5 mM MgCl_2_, and 1 *μ*M of each primer (Table 1). The program of PCR consisted of 94°C for 5 min, followed by 30 cycles of 94°C for 1 min, 55°C for 1 min, and 72°C for 1 min, with a final extension step at 72°C for 10 min.

### 2.5. Plasmids Profile

To analyze the plasmid profile, the plasmids of wild strains and the plasmids R27 (110 MDa), JPN11 (66 MDa), PSA (23 MD), and pRK (13, 2 MDa) used as markers of molecular mass were extracted by alkaline lysis [11]; the molecular weight of the plasmids was measured (LabImage 1D software) and the ability to transfer was determined.

The strains that harbored plasmids were chosen for mating experiments. The strains were grown in LB (Luria Bertani Broth) until the exponential phase. 1.2 mL of this culture was transferred to a flask containing 0.4 mL of the recipient culture in the stationary phase,* E. coli* K12-711 [12]. The mixture was incubated at 37°C for 3 hours. Transconjugants resistant to drugs were selected on MacConkey agar containing inhibitory concentrations of nalidixic acid (resistance present in* E. coli* K12-711), tetracycline, ampicillin, or kanamycin. The colonies grown on each selective plate were tested for the presence of virulence genes and pathogenicity islands, according to the virulence pattern of the donor strain. The resistance profile, necessary for this test, is shown in Table 2.

## 3. Results and Discussion

Extraintestinal pathogenic* Escherichia coli *(ExPEC) is one of the leading causes of bloodstream infections (BSI) worldwide. In Brazil, it was observed a high mortality associated with BSI [13]. Despite the importance of* E. coli* bloodstream infections due to their high morbidity and mortality, the pathogenesis is not well known [14, 15] and not studied enough in South America [4, 5].

In this study we screened 20 strains from bacteremia from newborns (10%), children aged between 6 months and 14 years (20%), and adults (70%) and from two Brazilian teaching hospitals for the determination of phylogenetic group, pathogenicity islands (PAI) associated sequences, and important virulence factors responsible for extraintestinal pathogenesis. The results were compared with those obtained with isolates from the stools of healthy humans. The relationship between the presence of PAIs, virulence genes, and the phylogenetic group was analyzed.

Among the 20 isolates from the hemocultures tested, 70% of the isolates displayed PAI associated sequences (total of 22 islands), while in the commensal strains 52.94% of them displayed PAI (total of 45 islands). In agreement with previously published data [7, 16, 17], PAI IV_536_ was the most prevalent in both groups, following the PAI I_CFT073_ and PAI II_CFT073_, as shown in Tables 3 and 4. PAI IV_536_ has been described by many researchers as a stable island, and it is one of the most commonly found PAIs in enterobacteria [7, 18]. Our results show that the islands present in UPEC, although poorly researched in septicemic strains, are also found in* E. coli* isolated from hemocultures. This similarity can be associated with the fact that the urinary tract infection is one of the most common infections and bacteraemia is often a complication of this infection. But there are other ways for the presence of bacteria in the blood, such as meningitis and polymicrobial intra-abdominal infections. The presence of bacteria in the bloodstream suggests the ability of these pathogens to survive in an environment with scarce free iron and to resist the bactericidal activity in the blood [4].

It is not clear yet if all* E. coli* from the intestinal tract of healthy people can be considered commensal, as some isolates showed up to five pathogenicity islands. Already it has been reported that ExPEC can asymptomatically colonize the intestinal tract [7].

In this study, genes related to toxin and hemolysin production were researched and included* cnf*2,* cnf*1,* hly*A,* hly*F, and* cva*C. The* hly*A gene is frequently detected in ExPEC and it was the most prevalent gene in our strains of hemoculture (30%) [19, 20].* Escherichia coli *hemolysin (*hly*A) is a pore-forming bacterial exotoxin that may contribute to the virulence of bacteria during bloodstream infection and sepsis [20, 21]. The PAIs I_536_, II_536_, I_CFT073_, I_J96,_ and II_J96_ harbor a copy of hlyABCD system encoding *α*-hemolysin, and in our study, six strains harbor* hly*A gene and three also had these PAIs (Table 3). Commensal strains showed a large prevalence of the* hly*A gene too, with 52.94% of the strains, and did not show a good correlation with the PAIs, since only 3 of 27 isolates that had the* hly*A had the respective PAI. Of the virulence genes encoding adhesins (*pap*C,* pap*G,* sfa*A,* sfa*S, and* afa*),* pap*C and* pap*G were the most prevalent in the strains of hemoculture. These two genes were present in 30% of our strains and were always found together in the isolates, including in commensal strains. These genes are part of the mannose-resistant P-fimbriae operon and have been associated with* E. coli* isolated from bacteremia [22, 23]. The PAIs I_J96_, I_CFT073,_ and II_CFT073_ harbor genes encoding P-fimbriae. Of the six strains of hemocultures containing the genes* pap*C and* pap*G, three contained corresponding PAIs (Table 3). Meanwhile, all the commensal strains containing the* pap*C and* pap*G genes also have PAI I_CFT073_, showing a good correlation between them.

Siderophore production is important for bacterial survival in the bloodstream. The aerobactin siderophore system is an important virulence factor that contributes to bacterial growth in host tissues and fluids where iron availability is limited [24, 25]. The aerobactin receptor (IutA) is commonly associated with extraintestinal* E. coli* and those isolated from bacteremia [22, 26]. In the present study,* iut*A was the most commonly found virulence gene, present in 65% of the isolates tested (Table 3), different from commensal which did not show this gene. PAI I_CFT073_ contains aerobactin genes, and 5 of the 13 strains containing the* iut*A gene also contained sequences similar to PAI I_CFT073_. Genes corresponding to other iron uptake systems were also identified:* iro*N, encoding the salmochelin receptor, was present in 55% of isolates; and* fyu*A, encoding the yersiniabactin receptor [24, 25], was present in 45% of isolates. PAI IV_536_ contains yersiniabactin encoding genes, and 8 of the 9 isolates exhibiting the* fyu*A gene contained PAI IV_536_ related sequences. Thus, there is a good correlation between the presence of this island and the genes encoding yersiniabactin. There was a good correlation between the commensal strains too and of the 24 isolates that contained the* fyu*A gene, 22 isolates had also PAI IV_536_.

Of the genes that confer serum resistance (*kps*II, K1,* tra*T, and* iss*),* tra*T, which encodes an outer membrane lipoprotein that contributes to serum resistance [27] was detected in 50% of the isolates.

The invasion determinant encoded by the* ibe*10 gene was present in 10% of the isolates, whereas the* omp*T gene, encoding an outer membrane protease, was present in 15% of the isolates.

These results demonstrate that the virulence of septicemic* E. coli* is not summarized by the presence of a single virulence factor, since each step in the infection process can be mediated by different virulence factors [14, 28], but the expression of the combination of virulence factors together with the imbalance between immune defenses and characteristics of the environment determines a multifactorial outcome [29].

Several studies have demonstrated that isolates belonging to phylogenetic group B2 are more commonly extraintestinal pathogenic strains [3, 15, 22]. Our results demonstrated that group B2 (40%) was the most common group among the* E. coli* strains from hemoculture (Table 4). Strains belonging to group B2 also had the most PAI associated sequences, and 15 of the total 22 PAIs identified were present in this group. In contrast, in the commensal strain the most prevalent group was A (72.54%) and this group had a greater number of PAIs (86.95%) (Table 4). Moreover, seven of eight strains of group B2 had PAIs, against only one of six strains of group A. However, despite reports in the literature that isolates belonging to groups A and B1 are more often strictly commensal strains from the intestinal microbiota [3, 7, 15, 22], ten of the isolates sampled belonged to groups A and B1 (Table 3), demonstrating that these groups are also capable of causing systemic infection. The results of the current study indicate that isolates that are phylogenetically characterized as mainly commensal can in some cases be isolated from bloodstream infections, reinforcing the concept that virulence is associated with the presence of multiple virulence factors and is dependent on the host's immune system [29]. The results also showed that group B2* E. coli*, despite being uncommon among commensal strains, can be present in intestinal flora (11.76% of our commensal strains) (Table 4), suggesting that they may act as a reservoir for bacteria that can cause extraintestinal infection [7].

In previous reports, ExPEC strains from group B2 have been shown to contain more virulence factors than those from groups A and B1 [15, 22, 30]. However, our strains showed on average 4 to 5 virulence factors genes, regardless of the phylogenetic group, and despite the correlation between the presence of virulence genes and strains belonging to phylogenetic group B2, some isolates from group B2 were found to have few virulence genes, whereas some isolates from groups A and B1 had up to 8 virulence genes, while some strains of group B2 with a greater number of virulence factors had 8 virulence genes too (Table 3). Thus, although some isolates from groups A and B1 are limited in their virulence gene content and are not likely to be highly virulent, others in these groups contained multiple virulence factors genes that could contribute to extraintestinal virulence.

Similarly, the virulence genes are not only associated with the PAIs, because some ExPEC also harbor virulence genes in plasmids [31]. Furthermore, PAI are located in regions of high genetic mobility, which show elements that allow recombination and, consequently, PAI rearrangement, deletion, and/or acquisition of foreign DNA [7].

Given the importance of plasmids as mobile elements in the horizontal gene transfer, the plasmid profile was investigated. In this study, 14 strains showed these plasmids, which ranged from 1 to 5 plasmids per strain, and three plasmids transferred to another* E. coli* by conjugation, appearing to be conjugative plasmids. The transconjugants (sample 5, 17, and 19) received genes* iut*A,* omp*T,* hly*F,* iro*N,* tra*T, and* iss*, showing the possible presence of these virulence genes in plasmids (Table 5). Thus, the presence of transferable plasmids in* E. coli* isolated from hemocultures can contribute to the horizontal transfer of virulence genes to nonpathogenic isolates and interestingly the conjugative plasmids 5.2, from strain 5, had* iss*,* iro*N,* omp*T, and* hly*F genes, whose genes are generally present in typical APEC plasmids in a conserved virulence plasmidic (CVP) region [32]. These findings support the idea that APEC or* E. coli* from commercial chicken carcasses has a potential zoonotic risk, as well as serves as a reservoir for virulence genes for ExPEC strains [9, 28, 33].

As ExPEC pathogenicity is due to genetic features such as virulence genes, pathogenicity islands, and plasmids associated with the virulence, the study of genetic factors is important to better understand these important pathogens. Thus, for the screening and even prevention of blood-borne diseases caused by ExPECs, further research should be conducted on the genetic features associated with the virulence of these pathogens. Although in stool samples of healthy people the presence of commensal strains is common, our results showed that the intestinal microbiota may harbor* E. coli* of phylogenetic group B2. Also* E. coli* with PAIs and virulence genes suggest that the intestinal microbiota may act as a reservoir of ExPEC with virulence genetic factors present at the* E. coli* from blood stream infection.

## Figures and Tables

**Table 1:** Primers for detection of PAIs markers, phylogenetic analysis, virulence genes, and their respective virulence factors.

Genes	Sequence (5′ to 3′)	Size of product (bp)	Virulence factors	Reference
**PAI I**_ 536_	TAA TGC CGG AGA TTC ATT GTC AGG ATT TGT CTC AGG GCT TT	1.800	*α*-Haemolysin, CS12 fimbriae, and F17-like fimbrial adhesin	Sabaté et al., 2006 [7]
**PAI II**_ 536 _	CAT GTC CAA AGC TCG AGC C CTA CGT CAG GCT GGC TTT G	1.000	*α*-Haemolysin and P-related fimbriae	Sabaté et al., 2006 [7]
**PAI IV**_ 536_	AAG GAT TCG CTG TTA CCG GAC TCG TCG GGC AGC GTT TCT TCT	300	Yersiniabactin siderophore system	Sabaté et al., 2006 [7]
**PAI I**_ CFT073_	GGA CAT CCT GTT ACA GCG CGC A TCG CCA CCA ATC ACA GC GAA C	930	*α*-Haemolysin, P-fimbriae, and aerobactin	Sabaté et al., 2006 [7]
**PAI II**_ CFT073_	ATG GAT GTT GTA TCG CGC ACG AGC ATG TGG ATC TGC	400	P-fimbriae and iron-regulated genes	Sabaté et al., 2006 [7]
**PAI I**_ J96 _	TCG TGC TCA GGT CCG GAA TTT TGG CAT CCC ACA TTA TCG	400	*α*-Haemolysin and P-fimbriae	Sabaté et al., 2006 [7]
**PAI II**_ J96 _	GGA TCC ATG AAA ACA TGG TTA ATG GG GAT ATT TTT GTT GCC ATT GGT TAC C	2.300	*α*-Haemolysin, Prs-fimbriae, and cytotoxic necrotizing factor 1	Sabaté et al., 2006 [7]
*chu*A	GAC GAA CCA ACG GTC AGG AT TGC CGC CAG TAC CAA AGA CA	279	Hemetransport in enterohemorrhagic O157:H7 *E. coli *	Clermont et al., 2000 [3]
*yja*A	TGA AGT GTC AGG AGA CGC TG ATG GAG AAT GCG TTC CTC AAC	211	Protein of function unknown	Clermont et al., 2000 [3]
TSPE4.C2	GAG TAA TGT CGG GGC ATT CA CGC GCC AAC AAA GTA TTA CG	152	Putative DNA fragment (TSPE4.C2) in *E. coli *	Clermont et al., 2000 [3]
*kps*II	GCG CAT TTG CTG ATA CTG TTG CAT CCA GAC GAT AAG CAT GAG CA	272	Capsule synthesis K1 e K5	Johnson and Stell, 2000 [8]
K1	TAG CAA ACG TTC TAT TGG TGC CAT CCA GAC GAT AAG CAT GAG CA	156	Capsule K1	Johnson and Stell, 2000 [8]
*cva*C	CAC ACA CAA ACG GGA GCT GTT CTT CCC GCA GCA TAG TTC CAT	680	Colicin V	Johnson and Stell, 2000 [8]
*iut*A	GGC TGG ACA TCA TGG GAA CTG G CGT CGG GAA CGG GTA GAA TCG	302	Aerobactin siderophore receptor	Johnson et al., 2008 [9]
*fyu*A	TGA TTA ACC CCG CGA CGG AA CGC AGT AGG CAC GAT CTT GTA	880	Yersiniabactin	Johnson and Stell, 2000 [8]
*pap*C	GAC GGC TGT ACT GCA GGG TGT GGC G ATA TCC TTT CTG CAG GCA GGG TGT GGC	328	P fimbriae	Le Bouguénec et al., 1992 [10]
*pap*G	CTG TAA TTA CGG AAG TGA TTT CTG ACT ATC CGG CTC CGG ATA AAC CAT	1070	P fimbriae	Johnson and Stell, 2000 [8]
*sfa*A	CTC CGG AGA ACT GGG TGC ATC TTA C CGG AGG AGT AAT TAC AAA CCT GGC A	410	Sfa fimbriae	Le Bouguénec et al., 1992 [10]
*sfa*S	GTG GAT ACG ACG ATT ACT GTG CCG CCA GCA TTC CCT GTA TTC	240	Sfa fimbriae	Johnson and Stell, 2000 [8]
*Afa *	GGC AGA GGG CCG GCA ACA GGC CCC GTA ACG CGC CAG CAT CTC	559	M fimbriae	Johnson and Stell, 2000 [8]
*ibe*10	AGG CAG GTG TGC GCC GCG TAC TGG TGC TCC GGC AAA CCA TGC	170	Invasion of brain endothelium	Johnson and Stell, 2000 [8]
*hly*A	AAC AAG GAT AAG CAC TGT TCT GGC ACC ATA TAA GCG GTC ATT CCC GTC	1.177	Hemolysin	Johnson and Stell, 2000 [8]
*cnf*1	AGG ATG GAG TTT CCT ATG CAG GAG CAT TCA GAG TCC TGC CCT CAT TAT T	498	Cytotoxic necrotizing factor 1	Johnson and Stell, 2000 [8]
*cnf*2	AAT CTA ATT AAA GAG AAC CAT GCT TTG TAT ATC TA	543	Cytotoxic necrotizing factor 2	Blanco et al., 1996 [34]
*tra*T	GGT GTG GTG CGA TGA GCA CAG CAC GGT TCA GCC ATC CCT GAG	290	Serum resistance	Johnson and Stell, 2000 [8]
*iro*N	AAT CCG GCA AAG AGA CGA ACC GCC T GTT CGG GCA ACC CCT GCT TTG ACT TT	553	Salmochelin siderophore receptor	Johnson et al., 2008 [9]
*omp*T	TCA TCC CGG AAG CCT CCC TCA CTA CTA T TAG CGT TTG CTG CAC TGG CTT CTG ATA C	496	Episomal outer membrane protease	Johnson et al., 2008 [9]
*hly*F	GGC CAC AGT CGT TTA GGG TGC TTA CC GGC GGT TTA GGC ATT CCG ATA CTC AG	450	Putative avian hemolysin	Johnson et al., 2008 [9]
*Iss *	CAG CAA CCC GAA CCA CTT GAT G AGC ATT GCC AGA GCG GCA GAA	323	Episomal increased serum survival	Johnson et al., 2008 [9]

**Table 2:** Plasmid and resistance profile of strains from hemocultures.

Strains	Plasmids	Molecular size of the plasmids (MDa)	Resistance profile
1	p1a	78	ApCbCfCmKnTr
2	p2a, b, c, d	113, 112, 86, 82	ApCbSmFoKnTr
3	p3a, b, c, d, e	112, 99, 78, 58, 29	ApCbCfCmSmFoGnKnSiSuTbTcTr
4	p4a	59	ApCfFoTcTr
5	p5a, b	99, 71	ApCbCfCmSmFoKnSuTcTr
6	p6a	112	ApCbCfCmSmFoGnKnSiTcTr
7	p7a	99	ApCbSmFoSu
8	NP	—	ApFo
9	NP	—	ApFo
10	p10a	82	ApCmFoTc
11	p11a	68	ApCbCmSmFo
12	NP	—	Ap
13	p13a, b, c, d, e	87, 68, 59, 50, 29	ApCbCfSmFoGnKnSuTbTcTr
14	p14a, b	62, 44	ApCbSm
15	NP	—	Ap
16	NP	—	Ap
17	p17a	87	ApCfCmSmKnTcTr
18	p18a	62	Ap
19	p19a	92	ApCbCmSmKnSuTcTr
20	NP	—	Ap

NP: no plasmid; Ap: ampicillin; Cb: carbenicillin; Cf: cephalothin; Cm: chloramphenicol; Sm: streptomycin; Fo: fosfomycin; Gn: gentamicin; Kn: kanamycin; Si: sisomicin; Su: sulfonamide; Tb: tobramycin; Tc: tetracycline; Tr: trimethoprim.

**Table 3:** Study of pathogenicity islands, phylogenetic classification, and virulence genes in 20 ExPEC strains from human hemocultures.

Strains	PC	Pathogenicity islands				Virulence genes															
		IV_536_	I_CFT073_	II_CFT073_	I_J96_	*kps*II	K1	*fyu*A	*pap*C	*pap*G	*sfa*A	*Ibe*10	*hly *	*cnf*1	*cnf*2	*tra*T	*iut*A	*iro*N	*omp*T	*hly*F	*iss *
1	B2	+	+	+	−	+	−	−	+	+	+	−	+	−	−	−	+	+	−	−	−
2	B2	+	+	+	−	+	+	−	−	−	−	−	−	−	−	−	+	+	−	−	−
3	B2	+	+	−	−	+	+	−	−	−	−	−	−	−	−	−	+	−	−	−	−
4	B2	−	−	−	+	+	+	−	−	−	−	+	−	−	−	−	+	−	−	−	−
5	A	−	−	−	−	−	−	−	−	−	−	−	−	−	−	+	−	+	+	+	+
6	A	−	−	−	−	−	−	−	+	+	−	−	−	−	−	+	+	+	−	−	−
7	B2	+	+	−	−	+	−	+	+	+	−	−	+	−	−	+	+	+	−	−	−
8	B2	+	+	−	−	−	−	−	−	−	−	−	−	−	−	−	−	−	−	−	−
9	B2	+	+	−	−	+	+	+	+	+	+	−	+	−	−	−	+	−	−	−	−
10	B1	+	−	−	−	−	−	+	−	−	−	−	+	−	+	+	−	+	+	+	+
11	A	+	−	−	−	−	−	+	−	−	−	−	+	−	−	+	+	+	−	−	−
12	B1	+	−	−	−	−	−	+	−	−	−	+	−	−	−	−	−	−	−	−	−
13	A	−	−	−	−	+	+	+	+	+	−	−	+	−	−	+	+	−	−	−	−
14	A	−	−	−	−	+	+	−	+	+	−	−	−	−	−	+	+	+	−	−	−
15	A	−	−	−	−	−	−	−	−	−	−	−	−	−	−	−	+	−	−	−	−
16	B2	−	−	−	−	−	−	−	−	−	−	−	−	−	−	−	−	+	−	−	−
17	D	+	−	−	−	−	−	+	−	−	−	−	−	−	−	+	+	−	−	−	−
18	B1	+	−	−	−	−	−	+	−	−	−	−	−	−	−	+	−	−	−	−	−
19	D	+	−	−	−	−	−	+	−	−	−	−	−	−	−	+	+	+	−	−	+
20	B1	−	−	+	−	−	−	−	−	−	−	−	−	−	−	−	−	+	+	−	+

PC: phylogenetic classification; pathogenicity islands: PAI IV_536_, PAI I_CFT073_, PAI II_CFT073_, and PAI I_J96_; adhesions: *pap*C, *pap*G, *sfa*A, *sfa*S, and *afa*; invasion: *ibe*10; toxins: *cnf*2, *cnf*1, *hly*A, *hly*F, and *cva*C; iron uptake systems: *fyu*A, *iut*A, and *iro*N; serum resistance: *kps* II, K1, *tra*T, and *iss*; proteases: *omp*T.

**Table 4:** Distribution of pathogenicity islands (PAI) according to phylogenetic classification (PC), among ExPEC and commensal strains.

PC	PAI I_536_	PAI II_536_	PAI IV_536_	PAI I_J96_	PAI I_CFT073_	PAI II_CFT073_	Total of PAIs	Total of strains
B2 (ExPEC)	0	0	6	1	6	2	15	8
B2 (commensal)	0	0	2	0	2	1	5	6
D (ExPEC)	0	0	2	0	0	0	2	2
D (commensal)	0	0	0	0	0	0	0	3
B1 (ExPEC)	0	0	3	0	0	1	3	4
B1 (commensal)	0	0	0	0	2	0	2	5
A (ExPEC)	0	0	1	0	0	0	1	6
A (commensal)	3	3	21	0	7	4	38	37

**Table 5:** Genetic characterization of ExPEC transconjugants.

Strains	*iss *	*tra*T	*iut*A	*iro*N	*omp*T	*hly*F
*E. coli* K12-711	−	−	−	−	−	−
*E. coli* 5	+	+	−	+	+	+
5.2	+	+	−	+	+	+
*E. coli* 17	−	+	+	−	−	−
17.1	−	+	+	−	−	−
*E. coli* 19	+	+	−	−	−	−
19.1	−	+	−	−	−	−

Serum resistance: *iss* and *tra*T; iron uptake systems: *iut*A and *iro*N; proteases: *omp*T; toxin: *hly*F.

Strains (donator of plasmids) = 5, 17, and 19; Tranconjugants = 5.2, 17.1, and 19.1.
